# Influence of Planting Density on Sweet Potato Storage Root Formation by Regulating Carbohydrate and Lignin Metabolism

**DOI:** 10.3390/plants12102039

**Published:** 2023-05-19

**Authors:** Qinggan Liang, Hongrong Chen, Hailong Chang, Yi Liu, Qinnan Wang, Jiantao Wu, Yonghua Liu, Sunjeet Kumar, Yue Chen, Yanli Chen, Guopeng Zhu

**Affiliations:** 1 Key Laboratory of Quality Regulation of Tropical Horticultural Crop in Hainan Province, School of Horticulture, Hainan University, Haikou 570228, China; 2 Institute of Nanfan & Seed Industry, Guangdong Academy of Sciences, Guangzhou 510310, China; 3 Hainan Yazhou Bay Seed Laboratory, Sanya Nanfan Research Institute of Hainan University, Sanya 572025, China

**Keywords:** sweet potato, carbohydrate, lignin, storage root number, yield

## Abstract

An appropriate planting density could realize the maximum yield potential of crops, but the mechanism of sweet potato storage root formation in response to planting density is still rarely investigated. Four planting densities, namely D15, D20, D25, and D30, were set for 2-year and two-site field experiments to investigate the carbohydrate and lignin metabolism in potential storage roots and its relationship with the storage root number, yield, and commercial characteristics at the harvest period. The results showed that an appropriate planting density (D20 treatment) stimulated cambium cell differentiation, which increased carbohydrate accumulation and inhibited lignin biosynthesis in potential storage roots. At canopy closure, the D20 treatment produced more storage roots, particularly developing ones. It increased the yield by 10.18–19.73% compared with the control D25 treatment and improved the commercial features by decreasing the storage root length/diameter ratio and increasing the storage root weight uniformity. This study provides a theoretical basis for the high-value production of sweet potato.

## 1. Introduction

Sweet potato (*Ipomoea batatas* L.) is the seventh most important crop around the world for its yield and cultivated area, and China is the largest sweet potato producer worldwide [1]. The yield of sweet potatoes is determined by the average storage root number per plant and the individual storage root weight [2]. The average storage root number per plant significantly contributes to the yield [3]. Improving the average storage root number per plant could lead to a good appearance and improve the commercial characteristics and yield of sweet potato storage roots [4]. Sweet potato storage root formation is a vital process determined by the degree of primary cambium development and stele cell lignification in adventitious roots [5]. The first clear sign of storage roots is the formation of secondary cambial cells (anomalous cambium) encircling the adventitious root’s primary and secondary xylem elements [6]. Moreover, cambial cell proliferation forms starch-accumulating parenchyma cells in the root’s vascular cylinder, accompanied by massive starch accumulation [7]. Simultaneously, carbohydrates provide energy for cambium cell development. The lignin content in potential storage roots is a common physiological indicator of sweet potato for the calculation of lignification [8]. The upregulation of carbohydrate metabolism and down-regulation of lignin biosynthesis usually facilitate sweet potato storage root formation [9]. Sucrose synthase (SuSy) catalyzes the reversible cleavage of sucrose into fructose and either uridine diphosphate glucose (UDP-G) or adenosine diphosphate glucose (ADP-Glc) [10]. SPS catalyzes the conversion of fructose-6-phosphate and uridine diphosphateglucose (UDP-glucose) into sucrose-6-phosphate [11]. AGPase catalyzes the first step of starch biosynthesis by producing ADP-Glc and pyrophosphate (PPi) from Glc-1-P and ATP [12]. SSS acts to elongate linear chains, SBE promotes chain branching, and GBSS is an enzyme that is responsible for the elongation of amylose chains [13]. According to Du [14] and Si [15], the SuSy and SPS activity in young roots has great potential to promote storage root formation and increase the storage root number in sweet potato.

Meanwhile, increased AGPase, SSS, SBE, and GBSS activity promoted starch synthesis and deposition in the sink, resulting in sink bulking [16]. Lignin biosynthesis was initiated with the deamination of phenylalanine by phenylalanine ammonialyase (PAL), followed by a series of reactions that involved numerous enzymes, such as cinnamate 4-hydroxylase (C4H), 4-coumarate: CoA ligase (4CL), p-hydroxycinnamoyl-CoA: quinate shikimate p-hydroxycinnamoyltransferase (HCT), caffeoyl-CoAO-methyltransferase (CCoAOMT), and cinnamyl alcohol dehydrogenase (CAD) [17]. C4H and 4CL are two enzymes involved in phenylpropane synthesis, and HCT catalyzes the reactions by converting coumaroyl-CoA into coumaroylshikimate/quinate and caffeoyl shikimate/quinate into caffeoyl CoA. CCoAOMT further catalyzes the reaction by methylating caffeoyl CoA to feruloyl CoA, and CAD catalyzes the final step of lignin biosynthesis by converting the corresponding cinnamyl aldehydes into cinnamyl alcohols [18,19,20]. The downregulation of gene expression in lignin biosynthesis could reduce the lignin content in the plant tissue, including the root and stem [8,21]. Ibkn1, Ibkn2, and Ibkn3 are members of the class I knotted 1-like (KNOX-box) gene family, and they are expressed in primary cambium cells [8,15]. They regulate cell proliferation and differentiation in potential storage roots [22,23], and their expression in storage roots was two-fold higher than that in fiber roots [8,15].

Plant root system development is significantly affected by the planting density. The root architecture’s response to planting density is mainly manifested in the alternation of the root length, root diameter, root biomass, and root number. In addition, this root system enables the plantlet to adapt to space and source competition by adjusting nutrition and water absorption [24]. A high planting density could cause crop logging by limiting lignin biosynthesis, decrease the root size, and limit the activity of root absorption for shoot development, ultimately causing a yield decline [24,25,26]. Under a low planting density, the source competition between individual plants could be alleviated, but the development between shoots and roots could lose its balance, which also limits yield formation [27]. Planting density is an important factor in regulating yield formation in the crop lifespan. However, the planting density of sweet potatoes is low in most areas of China at approximately 40,000–50,000 plants ha^−1^ (70–80 cm row space and 25–30 cm plant space). Therefore, increased planting density is a vital cropping measure to realize the maximum sweet potato yield potential [28,29]. An appropriate planting density is hypothesized to coordinate the relationship between shoot and root development and promote sweet potato storage root formation by stimulating carbohydrate accumulation and limiting lignin biosynthesis, ultimately increasing the storage root number. In the present study, two widely cultivated sweet potato cultivars, Yanshu 25 (YS-25) and Pushu 32 (PS-32), were used for 2-year and two-site field experiments. Four planting densities were set (D15, 15 cm plant distance and 83,280 plants ha^−1^; D20, 20 cm plant distance and 62,520 plants ha^−1^; D25, 25 cm plant distance and 50,025 plants ha^−1^; and D30, 30 cm plant distance and 41,640 plants ha^−1^; the row distance was set at 80 cm for all) to investigate carbohydrate and lignin metabolism in potential storage roots, cambium development, root morphology, the source–sink relationship during the storage root formation period, and the yield and its components. The storage roots’ commercial characteristics in the harvest period were also investigated. This work presents a theoretical foundation for improved sweet potato productivity and storage root commercial quality under a suitable planting density in China.

## 2. Results 

### 2.1. Sweet Potato Storage Root Yields, Yield Components, and Appearance Quality 

The results of the 2-year field experiment showed that YS-25 and PS-32 elicited the same effect of the planting density on the storage root yield, the yield and its components, and the appearance quality (Table 1). The storage root diameter and average storage root weight significantly increased with the decrease in planting density. Furthermore, the storage root yield significantly increased under a higher planting density (D15 and D20; *p <* 0.05) compared with the control D25 treatment. The yield increased by 1.56–5.45% and 3.46–9.87% under the D15 treatment and by 10.93–19.73% and 10.18–14.13% under the D20 treatment in 2021 and 2022, respectively. In addition, the average number of storage roots per plant increased initially and subsequently decreased as the planting density decreased. Compared with that under the control D25 treatment, the average storage root number per plant reached the peak value under the D20 treatment at a significant difference level (*p <* 0.05). The lowest CV and L/D ratio were also observed in the D20 treatment. 

### 2.2. Commercial Storage Root Characteristics

The results of the two-year field experiment depicted that the commercial storage root weight and large storage roots significantly increased with the reduction in planting density (*p <* 0.05). However, compared with the control D25 treatment, the D20 treatment significantly increased the commercial storage root number, primary medium storage root number, and commercial storage root yield (*p <* 0.05). In addition, the commercial storage root yields increased by 23.35–66.42% and 19.02–33.46% in 2021 and 2022, respectively (Table 2).

### 2.3. Storage Root Traits at Closure Period

During the canopy closure period, the 2-year field experiment showed a similar pattern of planting density on the storage root traits (Table 3). The average storage root weight, diameter, and mature storage root number significantly increased with the reduction in planting density (*p* < 0.05). Meanwhile, the storage root number per plant and the developing storage root number did not significantly increase at a higher planting density (D15 and D20) compared with those under the control D25 treatment (*p <* 0.05). Moreover, the developing storage root number was highest in the D20 treatment (*p <* 0.05). 

### 2.4. Effect on Root Development System and Potential Storage Root Traits

The planting density significantly affected sweet potato’s adventitious root formation and potential storage root development (Table 4). Compared with the control D25 treatment, the D20 treatment significantly improved the adventitious root number, potential storage root diameter, weight, and ratio of potential storage root weight during the storage root initiation stage (during 0–25 days after planting; *p* < 0.05). By contrast, these parameters slightly decreased or were similar to those at the lower planting density 35 days after planting. However, the total root fresh weight significantly increased with the decrease in planting density during the storage root formation period (*p* < 0.05).

### 2.5. Effect on Dry Biomass Accumulation and Allocation

A high accumulation and allocation ratio of dry weight in the root system benefits storage root formation. The results indicated that the total plant dry weight and the root and shoot dry weight significantly increased with the increment in the plant distance (*p* < 0.05, Table 5). However, the allocation ratio of root dry weight and the root/shoot ratio dramatically increased at a higher planting density (D15 and D20) and reached the peak values under the D20 treatment (*p* < 0.05). The allocation ratio of shoot dry weight had no significant difference among treatments at 15–25 days after planting (*p* > 0.05), but it significantly decreased under the D20 treatment at 35 days after planting (*p* < 0.05).

### 2.6. Influence on Expression of Genes Associated with Carbohydrate and Lignin Metabolism

**Figure 1:**
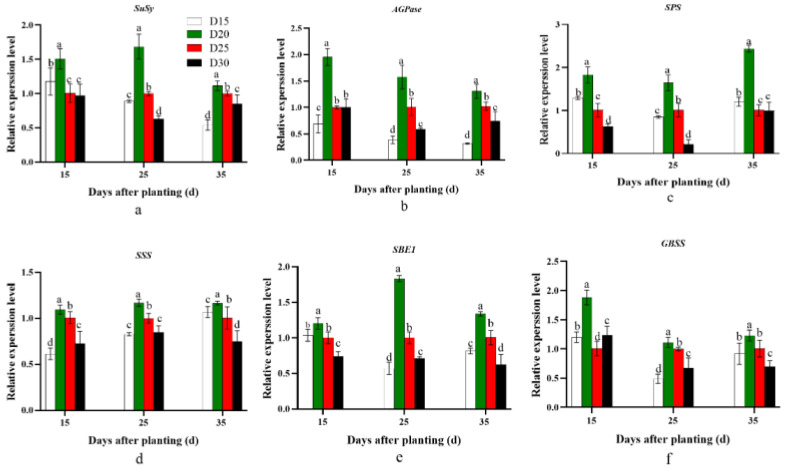
Effect of planting density on the expression of carbohydrate metabolism genes, namely *SuSy* (**a**), *AGPase* (**b**), *SPS* (**c**), *SSS* (**d**), *SBE1* (**e**), and *GBSS* (**f**), in YS-25 during storage root formation period (2022 Sanya). D15, 15 cm plant distance and 83,280 plants ha^−1^; D20, 20 cm plant distance and 62,520 plants ha^−1^; D25, 25 cm plant distance and 50,025 plants ha^−1^; D30, 30 cm plant distance and 41,640 plants ha^−1^. The row distance was set at 80 cm for all. 15 d, 15 days after planting; 25 d, 25 days after planting; 35 d, 35 days after planting. Error bars represent 1 SD (*n* = 3) within the same column, and different letters indicate a significant difference between treatments (*p* < 0.05).

The expression of genes’ regulating sucrose enzymolysis and starch synthesis had a similar pattern during the storage root formation period. The gene expression levels of *SuSy*, *AGPase*, *SPS*, *SSS*, *SBE1*, and *GBSS* in the D20 treatment were significantly enhanced compared with those in the control D25 treatment. Furthermore, a significant decrease in the expression of these genes was observed as the planting density decreased (*p* < 0.05; Figure 1).

**Figure 2:**
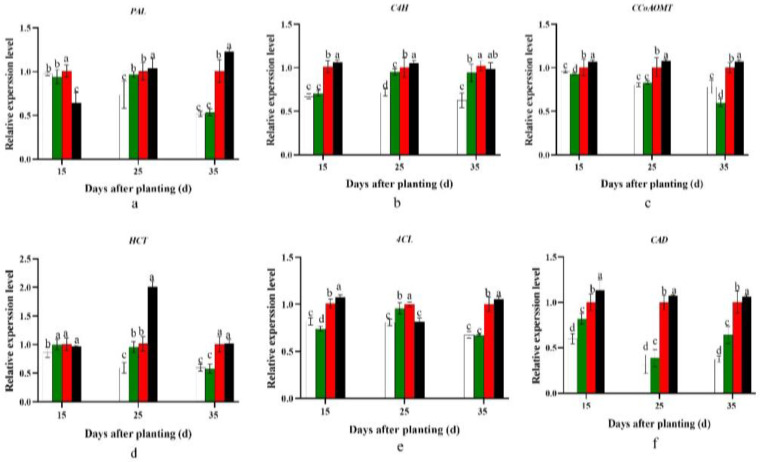
Effect of planting density on expression levels of lignin metabolism genes, namely *PAL* (**a**), *C4H* (**b**), *CCoAOMT* (**c**), *HCT* (**d**), *4CL* (**e**), and *CAD* (**f**), in YS-25 during storage root formation period (2022 Sanya). D15, 15 cm plant distance and 83,280 plants ha^−1^; D20, 20 cm plant distance and 62,520 plants ha^−1^; D25, 25 cm plant distance and 50,025 plants ha^−1^; D30, 30 cm plant distance and 41,640 plants ha^−1^. The row distance was set at 80 cm for all. 15 d, 15 days after planting; 25 d, 25 days after planting; 35 d, 35 days after planting. Error bars represent 1 SD (*n* = 3) within the same column, and different letters indicate a significant difference between treatments (*p* < 0.05).

Differences were observed in the expression of *PAL*, *C4H*, *CCoAOMT*, *HCT*, *4CL*, and *CAD*. The expression of these genes (referring to lignin biosynthesis) in the D20 treatment significantly decreased compared with that in the control D25 treatment (*p* < 0.05). However, a significant increase was observed with a further decrease in the planting density (*p* < 0.05; Figure 2).

### 2.7. Effect on Carbohydrate and Lignin Content

**Figure 3:**
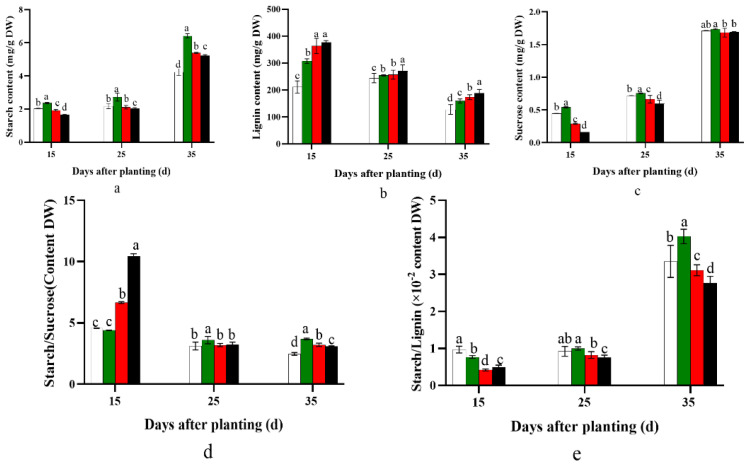
Effect of planting density on starch (**a**), sucrose (**b**), and lignin content (**c**), and ratios of starch/sucrose (**d**) and starch/lignin (**e**), in YS-25 during storage root formation period (2022 Sanya). D15, 15 cm plant distance and 83,280 plants ha^−1^; D20, 20 cm plant distance and 62,520 plants ha^−1^; D25, 25 cm plant distance and 50,025 plants ha^−1^; D30, 30 cm plant distance and 41,640 plants ha^−1^. The row distance was set at 80 cm for all. 15 d, 15 days after planting; 25 d, 25 days after planting; 35 d, 35 days after planting. Error bars represent 1 SD (*n* = 3) within the same column, and different letters indicate a significant difference between treatments (*p* < 0.05).

The starch and sucrose content in the potential storage root had the same effect. Compared with the control, the starch and sucrose content in the potential storage root significantly increased in the D20 treatment (*p* < 0.05) and decreased with a reduction in planting density. Furthermore, the increment in starch and sucrose content at 35 days after planting was more dramatic than at 15 and 25 days after planting (Figure 3a,b).

The lignin content in the potential storage root significantly increased with the reduction in planting density (*p* < 0.05), but the content in each treatment gradually decreased with the prolongation of the planting period (*p* < 0.05; Figure 3c). 

The ratios of starch to sucrose and starch to lignin showed a similar trend. The value was significantly higher in D20 than in the control at 25 and 35 days after planting (*p* < 0.05). Meanwhile, 15 days after planting, the ratio of starch to lignin was steadily reduced as the planting density decreased (*p* < 0.05), whereas the ratio of starch to sucrose showed the reverse trend (*p* < 0.05; Figure 3d,e). 

### 2.8. Influence on Genes Invloved in Cambium Development and Potential Storage Root Anatomy

**Figure 4:**
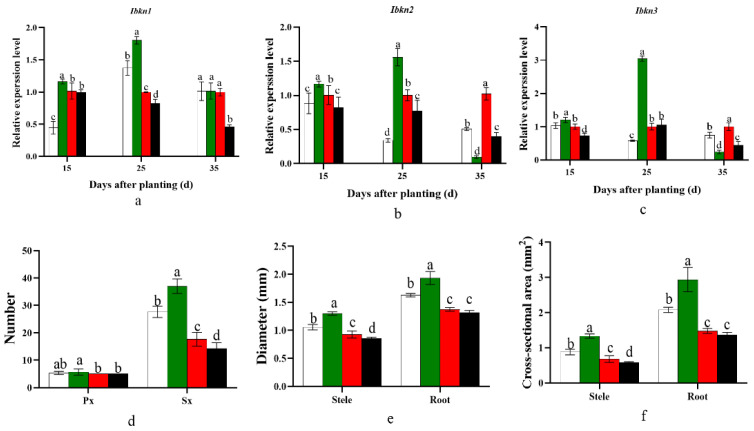
Effect of planting density on gene expression levels of *Ibkn1* (**a**), *Ibkn2* (**b**), and *Ibkn3* (**c**) during storage root formation period. Analysis of the potential storage root section anatomy, including the number of Px and Sx (**d**), the diameter of the stele and root (**e**), and the cross-sectional area of the stele and root (**f**) at 15 days after planting. D15, 15 cm plant distance and 83,280 plants ha^−1^; D20, 20 cm plant distance and 62,520 plants ha^−1^; D25, 25 cm plant distance and 50,025 plants ha^−1^; D30, 30 cm plant distance and 41,640 plants ha^−1^. The row distance was set at 80 cm for all. 15 d, 15 days after planting; 25 d, 25 days after planting; 35 d, 35 days after planting. Error bars represent SD (*n* = 3) within the same column, and different letters indicate a significant difference between treatments (*p* < 0.05).

**Figure 5:**
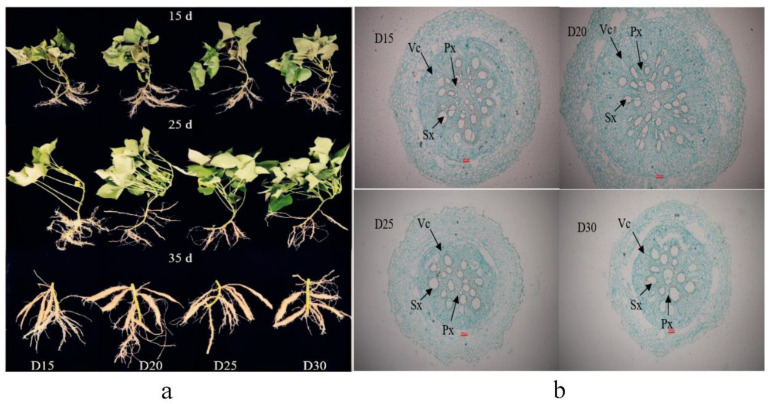
Effect of planting density on sweet potato YS-25 root growth and development during storage root formation period (**a**) and cambium development at 15 days after planting (**b**). D15, 15 cm plant distance and 83,280 plants ha^−1^; D20, 20 cm plant distance and 62,520 plants ha^−1^; D25, 25 cm plant distance and 50,025 plants ha^−1^; D30, 30 cm plant distance and 41,640 plants ha^−1^. The row distance was set at 80 cm for all. 15 d, 15 days after planting; 25 d, 25 days after planting; 35 d, 35 days after planting. Px, protoxylem; Sx, secondary xylem; Vc, vascular cambium. Scale bar = 100 μm.

*Ibkn1*, *Ibkn2*, and *Ibkn3*, which regulate potential storage root cambium development, were significantly higher in the D20 treatment than in the control D25 treatment at 0–25 days after planting. However, the expression of *Ibkn2* and *Ibkn3* significantly decreased 25–35 days after planting, and *Ibkn1* was similar to the control D25 treatment. Furthermore, the gene expression levels were significantly reduced when the planting density was further decreased (Figure 4a–c). Consequently, the D20 treatment significantly enhanced the number of protoxylems and secondary xylem, the diameter of the stele and potential root, and the cross-sectional area of the stele and potential storage root 15 days after planting compared with the control D25 treatment (Figure 4d–f and Figure 5).

### 2.9. Correlation Analysis of Storage Root Number Per Plant, Fresh Weight, and Yield with Relative Gene Expression

The correlation analysis demonstrated that the storage root number per plant was positively correlated with *SuSy*, *SPS* (*p* < 0.05), *SSS*, *AGPase*, *GBSS* (*p* < 0.01), and *SBE*, which are involved in carbohydrate metabolism. Furthermore, no significant positive correlation was found in *PAL*, *4CL*, *C4H*, *CCoAOMT*, *CAD*, and *HCT*, which are involved in lignin biosynthesis, whereas *C4H* and *HCT* were found to be negatively correlated. *Ibkn1*, *Ibkn2,* and *Ibkn3,* which regulate cambium development, were also positively correlated with the storage root number per plant but with no significant difference (Table 6). The storage root fresh weight was insignificantly negatively correlated with all genes (*p* > 0.05; Table 6), and the yield was positively correlated with the genes involved in carbohydrate metabolism and cambium development and almost negatively correlated with the genes involved in lignin biosynthesis (Table 6).

## 3. Discussion

### 3.1. Effect of Planting Density on Carbohydrate and Lignin Metabolism and Cambium Cell Development

The upregulation of starch biosynthesis and downregulation of lignin biosynthesis in potential storage roots are the main events during the storage root formation period [8,15,22], probably because the activity of carbon flow towards carbohydrate metabolism is stronger than the delivery into phenylpropanoid biosynthesis during the storage root formation period [14,30]. Previous findings have shown that low sucrose and high hexose content in the root system could facilitate storage root formation [14,31]. This process could be explained by the fact that sucrose, the primary type of carbohydrate, is delivered from the source leaves to the root through the phloem, where it is subsequently broken down into hexoses to supply energy and the carbon skeletons needed for cell growth and root swelling. Sucrose is decomposed into hexoses by the SuSy and sucrose invertase pathways, and the former is the main source for fructose and UDP-glucose biosynthesis, which provides the substrate for starch biosynthesis [14,31]. Crop root development is adversely correlated with lignin deposition, and lignin accumulation has a negative effect on root development [8,32].

Furthermore, lignin and cellulose content have a negative correlation with starch accumulation in cassava tuber root formation [33,34]. Lignin content is a common physiological indicator of sweet potato lignification, which strongly inhibits sweet potato storage root formation [8,33,34]. In the present study, compared with the control D25 treatment, the D20 treatment significantly promoted sucrose and starch biosynthesis by significantly increasing the expression levels of *SuSy*, *SPS*, *SSS*, *AGPase*, *SBE1*, and *GBSS* (Figure 1). By contrast, it inhibited lignin biosynthesis by dramatically decreasing the *PAL*, *C4H*, *CCoAOMT*, *HCT*, *4CL*, and *CAD* expression levels (Figure 2). In addition, the ratios of starch to sucrose and starch to lignin were significantly higher in D20 than in the control D25 treatment at 15–35 days after planting, and significantly higher or lower at 0–15 days after planting, respectively (Figure 3). Previous studies revealed that sweet potato storage root formation was determined by stele cell lignification and cambium proliferation [5,6]. Furthermore, only the appearance of anomalous cambium could prevent stele lignification and favor storage root formation [33,34]. The KNOX1 protein stimulates cell differentiation by negatively regulating lignin biosynthesis [35]. *Ibkn1*, *Ibkn2*, and *Ibkn3* belong to the KNOX gene family, and their expression in storage root was two-fold higher than that in fiber roots [36]. In the present study, the D20 treatment promoted primary cambium cell development by upregulating *Ibkn1*, *Ibkn2*, and *Ibkn3* expression 0–25 days after planting (Figure 4). Therefore, 15 days after planting, the D20 treatment significantly increased protoxylems and secondary xylem, the stele and potential storage root diameter, and the stele and potential storage root cross-sectional area compared with the control D25 treatment. The correlation analysis found that the genes involved in carbohydrate metabolism were positively correlated with the storage root number per plant, and *SPS* and *GBSS* reached a significant level (*p* < 0.05 and *p* < 0.01, respectively). However, no significant positive correlation was found with the lignin biosynthesis genes, and *C4H* and *HCT* were negatively correlated (Table 6). Furthermore, the storage root fresh weight was negatively correlated with all genes’ expression, and the yield was positively correlated with the genes involved in carbohydrate metabolism and cambium development and almost negatively correlated with the genes involved in lignin biosynthesis. The correlation analysis strongly improved the theoretical understanding of these processes; specifically, upregulated carbohydrate and downregulated lignin biosynthesis could improve the yield by promoting storage root formation in sweet potatoes. 

### 3.2. Effect of Planting Density on Plant Dry Matter Dynamic and Root Development

Coordinating the shoot and root relationship is a considerable factor in promoting storage root formation. The formation of sweet potato storage roots comprises four main events: the initiation of cambial cells, cell division, cell expansion and growth, and carbohydrate storage [36,37]. Major developmental activities strongly rely on dry matter accumulation in roots. The current study indicated that the total plant dry matter significantly increased with the decrease in planting density. Furthermore, compared with the control D25 treatment, the D20 treatment significantly increased the root dry weight, root dry weight ratio, and R/T ratio. By contrast, the shoot dry matter weight significantly decreased during the storage root formation period. However, the shoot dry matter ratio was similar 0–25 days after planting and it dramatically decreased 35 days after planting (Table 5). Root development is primarily measured by the number, weight, diameter, and length of the roots, among other parameters, and these roots morphology could indicate environmental changes, such as stress, the degree of nutrition and water uptake, and, more crucially, the strength of root differentiation in sweet potatoes [38]. The number of adventitious roots controls the quantity of storage roots during the early growth stage; the more adventitious roots, the more storage roots that form [15,38]. The root diameter is an important parameter to indicate the degree of root thickening caused by root cambium cell division and differentiation. An adventitious root with a length of more than 20 cm could facilitate storage root formation [39]. The results of the present study indicated that an appropriate planting density under the D20 treatment significantly increased the adventitious root number but decreased the total root fresh weight. The potential storage root parameters, namely the potential storage root diameter and potential storage root weight, significantly increased in the D20 treatment at 0–25 days after planting and decreased at 25–35 days after planting compared with those in other treatments. Furthermore, the potential storage root weight ratio was always significantly higher in the D20 treatment than in other treatments (Table 4). 

### 3.3. Effect of Planting Density on Storage Root Yield, Components, and Commercial Characteristics

Altering the planting density is an effective practice in cereal crop production, balancing the relationship between the kernel number per spike and the average weight per hundred kernels [40]. A higher planting density seems inclined to support kernel formation while reducing the average weight per hundred kernels [41]. The maximum output from cereal crops could only be reached by balancing the number of kernels per spike and the average weight of a hundred kernels. Sweet potato storage root yields were determined by the average storage root number per plant and average storage root weight, and the storage root per plant was the most significant contributing factor to the yield [15,38]. An increased storage root number could lead to a good appearance and improve the commercial traits of this crop [38]. The canopy closure period is a key factor in determining the storage root number of sweet potatoes, and the storage root number is known at this point [15,38]. Hence, the maximum storage root number at the canopy closure period could result in a high sweet potato yield. The current study showed that an appropriate planting density (D20 treatment) could significantly increase the storage root number (mainly the developing storage root number) compared with the control D25 treatment.

By contrast, the storage root weight, mature storage root number, and storage root diameter remarkably increased during the canopy closure period as the planting density decreased (Table 3). Therefore, compared with the control D25 treatment, the appropriate planting density under the D20 treatment significantly increased the yield by dramatically increasing the storage root number per plant, instead of the storage root diameter and average storage root weight. As a result, it improved the storage roots’ commercial characteristics by significantly increasing the number of commercial and middle-sized storage roots and reducing the storage root weight CV and L/D ratio to create a good-quality appearance (Table 1 and Table 2).

## 4. Materials and Methods

### 4.1. Materials

Orange-fleshed and widely cultivated sweet potato cultivars in China, YS-25 and PS-32, with approximately four storage roots, were selected for this experiment. Vegetative terminal cuttings with the following characteristics were used: the length was approximately 25 cm, the excess buds and leaves were removed, and the top three fully unfolded leaves were retained. The cutting base was soaked with 30 mg kg^−1^ carbendazim for 5 min. In this experiment, the fertilizers were urea (46% N, Sinopec, Co., Ltd., Dongfang, China), calcium superphosphate (16% P_2_O_5_, SDIC Xinjiang Lop Nur Potassium Salt Co., Ltd., Hami, China), and potassium sulfate (52% K_2_O, Guangdong Zhanhua Group Co., Ltd., Zhanjiang, China).

### 4.2. Methods

#### 4.2.1. Experimental Design

The two-year and two-site field experiments were conducted from 2020 to 2022. The first round of field experiments was arranged on 1 November 2020 and plants were harvested on 1 March 2021. The second round was conducted on 5 November 2021 and plants were harvested on 5 March 2022. The China Meteorological Data Service Center provided the two growth seasons’ climate data, as shown in Table S1.

The first field experiment was carried out at the agricultural base of Hainan University, Haikou, China (20°06′ N, 110°33′ E), and the second was conducted at the research base of the Institute of Nanfan & Seed Industry, Guangdong Academic of Sciences at Yazhou District, Sanya, China (18°21′30′′ N, 109°9′54′′ E). The soil type of the two fields was sandy loam. All the soil physical and chemical properties at the 0–30 cm soil tillage layer are presented in Table S2. 

The field experiments were carried out as a two-factor split-plot design with five replicates in a randomized block arrangement. The two sweet potato cultivars, YS-25 and PS-32, were assigned to the main plot with four plant distances, namely D15, 15 cm plant distance and 83,280 plants ha^−1^; D20, 20 cm plant distance and 62,520 plants ha^−1^; D25, 25 cm plant distance and 50,025 plants ha^−1^; and D30, 30 cm plant distance and 41,640 plants ha^−1^. The row distance was set at 80 cm for all. Each sub-plot had five ridges covering 12.60, 16.80, 21.00, and 25.20 m^2^. Before planting, each treatment was initiated with 120 kg ha^−1^ N, 112 kg ha^−1^ P_2_O_5_, and 240 kg ha^−1^ K_2_O as a base fertilizer. 

Stem cuttings were planted with four nodes introduced into the soil by the oblique planting method. During the experimental period, the field management, including pest, disease, and weed control, consisted of local high-yield field practices.

#### 4.2.2. Sampling and Measurements

Ten representative plants from each treatment were divided into two groups evenly at 15, 25, and 35 days after planting. In one group, the six thickest roots from five plants were selected as potential storage roots [15,38], and the potential storage roots were washed with distilled water. Then, they were dried with a tissue, immersed in liquid nitrogen, and stored at −80 °C. These samples were obtained to analyze the gene expression. The root and shoot systems of five plants from the other group were separated. Each plant’s root system was examined to count the number of adventitious roots, weigh the total roots and potential storage root by using an electric weighing balance, and measure the potential storage root’s thickest part’s diameter by using a Vernier caliper. The thickest part, with approximately a 1.00 cm length of the potential storage root, was immersed in 70% FAA fixative solution (Scientific Phygene) to observe cambium development. The shoots and roots of each plant were blanched at 105 °C for 30 min and 80 °C to a constant weight by oven drying. The root dry matter was preserved to quantify the lignin and carbohydrate content.

Meanwhile, several parameters were obtained as follows:
Root/shoot ratio = root dry weight/shoot dry weight × 100%;
Ratio of root dry biomass allocation = root dry weight/total plant dry biomass × 100%;
Ratio of shoot dry biomass allocation = shoot dry biomass/total plant dry biomass × 100%;
Potential SR weight ratio = potential storage fresh weight/total root fresh weight × 100%.

At 45 days after planting, the division method of Wang [23] was used to calculate the potential storage roots weighed and the number per plant (Φ > 2.00 mm). Furthermore, the number of young storage roots (2.00 < Φ ≤ 5.00 mm), developing storage roots (5 < Φ ≤ 20 mm), and mature storage roots (Φ > 20 mm) was calculated. All store roots were harvested 120 days after planting, and the total weight of the fresh storage roots in each plot was recorded. Then, the average storage root numbers per plant, weight, and yield were calculated. Moreover, five representative plants per plot were selected to measure the diameter and length of the storage root, and then the length/diameter ratio (L/D ratio) was calculated [42]. The L/D ratio has been used to describe the shape in agricultural products (if an object has L/D ratio = 1, it is considered circular). The uniformity of the storage root weight is expressed by the coefficient of variation (CV), where CV = standard deviation/average. The smaller the CV, the better the uniformity of the storage root weight. In accordance with the grading standard of fresh sweet potato introduced by Si [38] and the actual production in China in recent years, the storage roots (Φ > 1.00 cm, FW > 50 g) were considered commercial storage roots. They were divided into large commercial storage roots (FW of 250–500 g), medium-sized commercial storage roots (FW of 100–250 g), and small commercial storage roots (FW 50~100 g). The number of commercial storage roots of each grade was investigated manually.

#### 4.2.3. Carbohydrate Content Determination

Sucrose and starch content was analyzed by anthrone colorimetry [43]. Approximately 200 mg of dry root sample was crushed into a powder and placed in a 10 mL centrifugal tube with 5 mL distilled water. Then, the sample was centrifuged at 3000 rpm for 5 min. The supernatants were collected in a 50 mL volumetric flask, and then distilled water was added to the scale mark. This sample was noted as solution A. The residue was dissolved by 10 mL of 3 mol mL^−1^ HCl and placed in a 50 mL volumetric flask. Then, it was placed in a water bath for 40 min, cooled down to room temperature, and combined with 10 mL of 3 mol mL^−1^ NaOH. Afterwards, distilled water was added to the scale mark, and 2 mL of this sample was transferred to a new 50 mL volumetric flask. Distilled water was added to the scale mark, and this sample was noted as solution B. Solution A was used for sucrose determination, and solution B was used for starch determination.

Approximately 2 mL of 2 mol L^−1^ KOH solution was transferred to 10 mL of solution A, boiled for 10 min, cooled down to room temperature, and diluted with distilled water to 50 mL. Afterwards, 2 mL of this sample was transferred to a new tube to react with the anthrone reagent. The sucrose content was measured spectrophotometrically at 640 nm. A sucrose solution (0.1%) was used as a standard solution.

Approximately 2 mL of solution B was transferred to the anthrone reagent and placed in a boiling water bath for 5 min. Then, it was cooled to room temperature, and the starch content was determined at 640 nm. Furthermore, a glucose solution (0.1%) was used to generate a standard curve.

#### 4.2.4. Lignin Content Determination

A lignin content assay kit was used to measure the lignin content in the potential storage root (Suzhou Comin Biotechnology Co., Ltd., Suzhou, China). Dried roots (15 mg) were crushed and sieved (0.25 mm). Each sample was transferred to a 10 mL stoppered glass test tube, and each tube was immersed with 1000 µL of reagent 1 and 40 µL of perchloric acid and then placed at 80 ℃ for 40 min. Then, 5 mg quartz sand per tube for three repetitions was used as the control. Each tube was shaken for 10 min. Then, 1000 µL of reagent 2 was added and mixed well. The mixture was centrifuged at 5000 rpm for 2 min, and then 40 µL of the supernatant was removed and mixed with 1960 µL of reagent 3. Subsequently, the mixture was evaluated at 280 nm. Moreover, the lignin content was calculated as follows:

Lignin content (mg/g dry weight) = (ΔA − 0.0068) ÷ 0.0694 × Vt × 10 − 3 ÷ W × T,

where ΔA = Asample − Actrl; Vt = total reaction system volume; W = sample weight; and T = dilution ratio.

#### 4.2.5. Root Anatomical Observation and Histochemical Analysis

Root samples were soaked in a 70% FAA fixation solution (FTY. Phygene Life Sciences Co., Ltd., Fuzhou, China) and dehydrated using an ethanol dilution series before being embedded in paraffin wax. Three samples from each treatment were cut into 15-μm-thick sections with a microtome (Campden Instruments Ltd., London, UK). They were deparaffinized in a histoclear solution and rehydrated with an ethanol dilution series to further prepare root sections for histochemical staining and autofluorescence imaging. Safranin–fast green staining was used to investigate the root vascular system. The deparaffinized samples were stained in safranin-O (1%) for 2 h, the excess dye was washed off with distilled water, and then the samples were stained in fast green (0.5%) for 10 s. Images were observed and captured by a Nikon DS-Fi1 digital camera. The number of protoxylems and secondary xylem elements, the stele diameter, and the stele cross-sectional area were counted or measured.

#### 4.2.6. Real-Time Quantitative PCR Performance 

The total RNA of potential storage roots was extracted in accordance with the Plant Total RNA Isolation Kit Plus (Foregene, RE05024, Chengdu, China). The quality of isolated RNA was described by the RNA concentration and strip integrity measured by a micro-spectrophotometer (UV–Vis spectrophotometer Q5000, Quawell Technology, Inc., Sunnyvale, CA, USA). The first-strain cDNA was generated by the MonScriptTM RTIII ALL-in-One with dsDNase (One-Step) (Monad Biotech Co., Ltd., Wuhan, China). Real-time quantitative PCR was performed in a 20 μL reaction volume containing 1× MonAMPTM ChemoHS qPCR Mix (Monad Biotech Co., Ltd., Wuhan, China). The procedures of real-time quantitative PCR were performed in two steps as follows: initiated with predenaturation at 95 °C for 10 min, followed by 40 cycles at 95 °C for 15 s and 60 °C for 30 s, and default settings were used to collect the melt curves. Quantitative analysis was conducted using the ABI QuanStudioTM 5 System with standard mode. qRT-PCR detection was performed in three biological replicates. The relative expression levels were estimated using the 2^−∆∆Ct^ method. β-*actin* was used as the internal control. The primers used in qRT-PCR are listed in Table S3.

#### 4.2.7. Statistical Analysis

Two-way ANOVA was applied to determine the statistical significance, with a significance level at *p* < 0.05, which was tested by LSD. The statistical analysis was performed using the SPSS software (Version 19), and the figures were designed using the GraphPad Prism software (Version 8.4.2 for Windows).

## 5. Conclusions

In this study, the response mechanism of the storage root number, yield, and storage root commercial traits to the planting density was explained from the aspects of carbohydrate and lignin metabolism, such as carbohydrate content, lignin content, and the related regulatory gene expression in potential storage roots, as well as the potential storage root histochemical analysis, plant dry matter dynamics, and young root traits. The results proved that an appropriate planting density (D20 treatment in this study) could promote carbohydrate accumulation and inhibit lignin biosynthesis in potential storage roots. This finding could facilitate storage root formation to increase the storage root number by stimulating cambium cell division and inhibiting stele cell lignification. The planting density is an important factor in regulating carbohydrate and lignin metabolism in potential storage roots, affecting the number and yield and the commercial traits of sweet potato storage roots. However, storage root formation and storage root bulking are two important events that determine the final yield of this crop. Therefore, the findings were confirmed by taking into account the storage root bulking mechanism and photosynthetic features in response to planting density during the storage root bulking period (50–90 days after planting, data not given in this study).

## Figures and Tables

**Table 1:** Effect of planting density on storage root yield, yield components, and appearance quality.

Years	Cultivar	Treatment ^†^	SR Diameter (mm)	L/D Ratio	Average SR Weight (g)	CV (%)	Average SR Number Per Plant	Yield (kg·hm^−2^)	Yield Increment (%) ^‡^
2021 (Haikou)	YS-25	D15	36.95 b	2.7	104.56 d	5.64	3.33 c	29,017.72 b	5.45
		D20	38.75 a	2.5	111.13 c	1.77	4.67 a	33,030.38 a	19.73
		D25	39.39 a	2.6	127.26 b	11.87	4.33 ab	27,565.56 c	-
		D30	39.15 a	3.0	133.97 ab	5.49	4.33 ab	24,154.95 e	−14.11
	PS-32	D15	32.00 c	2.6	103.94 d	11.37	3.00 d	25,968.37 d	1.56
		D20	34.09 bc	2.5	113.57 c	5.74	4.00 b	28,401.60 bc	10.93
		D25	39.09 a	2.7	127.82 b	6.76	4.00 b	25,576.78 d	-
		D30	38.56 a	2.9	141.53 a	7.47	3.67 bc	21,628.44 f	−18.51
ANOVA									
	C		7.22 *	-	2.46 *	-	2.78 ^ns^	10.10 **	-
	T		5.21 **	-	3.43 *	-	3.07 *	11.39 ***	-
	C × T		1.66 ^ns^	-	0.93 ^ns^	-	0.41 ^ns^	0.47 ^ns^	-
2022 (Sanya)	YS-25	D15	38.04 c	2.6	88.50 d	7.69	5.26 bc	38,795.60 b	3.46
		D20	39.81 b	2.3	110.81c	2.90	6.19 a	42,883.33 a	14.13
		D25	39.72 b	2.4	131.66 b	6.71	5.71b	37,494.90 bc	-
		D30	42.71 a	2.7	170.61a	7.64	5.01 c	35,592.04 c	−5.34
	PS-32	D15	36.68 c	2.4	91.91 d	5.00	4.67 d	35,569.17 c	9.87
		D20	38.14 b	2.3	99.40 cd	2.06	5.76 b	35,795.45 c	10.18
		D25	38.97 b	2.6	131.42 b	4.40	4.93 c	32,437.80 d	-
		D30	40.47 b	3.1	160.09 a	6.61	4.83 cd	32,197.00 d	−0.62
ANOVA									
C			3.95 ^ns^	-	1.65 ^ns^	-	9.27 **	10.63 **	-
T			3.14 *	-	86.25 ***	-	10.11 **	12.36 **	-
C × T			8.36 ^ns^	-	1.12 ^ns^	-	0.61 ^ns^	0.56 *	-

Note: SR, storage root; Y, year; C, cultivar; T, treatment; YS-25, Yanshu25; PS-32, Pushu32. Two-way ANOVA, LSD. Values followed by different letters present significant differences among planting density treatments (*p <* 0.05). * *p <* 0.05; ** *p <* 0.01; *** *p <* 0.001; ^ns^, no significance. ^†^ D15, 15 cm plant distance and 83,280 plants ha^−1^; D20, 20 cm plant distance and 62,520 plants ha^−1^; D25, 25 cm plant distance and 50,025 plants ha^−1^; D30, 30 cm plant distance and 41,640 plants ha^−1^. The row distance was set at 80 cm for all. ^‡^ Compared with the D25 control treatment.

**Table 2:** Effect of planting density on storage root commercial characteristics at harvest period.

Years	Cultivars	Treatment ^†^	Commercial SR Weight Per Plant (g)	Commercial SR Number Per Plant	Large SR Number	Medium SR Number	Small SR Number	Commercial SR Yield (kg·hm^−2^)	Increment (%) ^‡^
2021 (Haikou)	YS-25	D15	122.61 b	2.33 d	0.33 c	1.67 c	0.33 a	19,558.23 b	−0.51
		D20	129.91 b	3.00 b	1.00 b	2.00 b	-	24,367.17 a	23.35
		D25	158.45 a	2.33 d	1.33 a	1.00 e	-	19,697.51 b	-
		D30	162.41 a	2.67 c	1.33 a	1.33 d	-	16,998.00 c	−16.16
	PS-32	D15	115.20 c	2.00 e	1.00 b	1.00 e	-	19,202.65 b	43.28
		D20	119.26 c	3.33 a	1.00 b	2.33 a	-	22,368.40 a	66.42
		D25	124.90 b	2.33 d	1.00 b	1.33 d	-	13,377.52 d	-
		D30	133.31 b	2.33 d	1.33 a	0.67 f	0.33 a	12,952.40 d	−3.24
ANOVA									
C			13.67 **	0.17 ^ns^	0.00 ^ns^	0.80 ^ns^	0.25 ^ns^	4.61 ^ns^	-
T			8.33 **	4.61 *	0.67 ^ns^	2.93 ^ns^	2.92 ^ns^	5.35 *	-
C × T			5.21 ^ns^	0.61 ^ns^	1.33 ^ns^	7.20 **	1.58 ^ns^	0.40 ^ns^	-
2022 (Sanya)	YS-25	D15	98.89 bc	4.00 c	-	2.00 c	2.00 a	33,272.02 a	29.60
		D20	117.89 bc	4.67 b	0.67 b	3.00 a	1.00 c	34,342.08 a	33.46
		D25	136.87 b	4.00 c	0.33 c	2.00 c	1.67 b	25,676.50 de	-
		D30	201.50 a	3.00 e	1.00 a	1.33 d	0.67 d	25,198.06 e	−1.95
	PS-32	D15	92.96 c	3.67 cd	-	1.67 c	2.00 a	28,412.07 c	5.97
		D20	102.05 bc	5.00 a	0.33 c	3.00 a	1.67 b	31,899.79 b	19.02
		D25	135.20 b	4.00 c	0.33 c	2.67 b	1.00 c	26,851.42 d	-
		D30	191.99 a	3.33 d	1.00 a	2.00 c	0.33 e	26,533.47 d	−1.13
ANOVA									
C			0.88 ^ns^	0.40 ^ns^	0.25 ^ns^	3.00 ^ns^	0.25 ^ns^	0.13 ^ns^	-
T			26.02 ***	7.07 **	6.25 **	17.2 ***	13.58 ***	7.23 **	-
C × T			0.12 ^ns^	0.13 ^ns^	0.25 ^ns^	3.00 ^ns^	2.92 ^ns^	0.41 ^ns^	-

SR, storage root; Y, year; C, cultivar; T, treatment; YS-25, Yanshu25; PS-32, Pushu32. Two-way ANOVA, LSD was used. Values followed by different letters present significant differences among planting density treatments (*p* < 0.05). * *p* < 0.05; ** *p* < 0.01; *** *p* < 0.001; ^ns^, no significance. ^†^ D15, 15 cm plant distance and 83,280 plants ha^−1^; D20, 20 cm plant distance and 62,520 plants ha^−1^; D25, 25 cm plant distance and 50,025 plants ha^−1^; D30, 30 cm plant distance and 41,640 plants ha^−1^. The row distance was set at 80 cm for all. ^‡^ Compared with the D25 control treatment.

**Table 3:** Effect of planting density on storage root traits at canopy closure period.

Years	Cultivar	Treatment ^†^	SR Weight (g)	SR Number Per Plant	Young SR Number (2 < Φ < 5 mm)	Developing SR Number (5 < Φ < 20 mm)	Mature SR Number (Φ > 20 mm)	SR Diameter (mm)
2021 (Haikou)	YS-25	D15	1.35 f	4.67 b	2.33 a	2.33 c	-	5.14 d
		D20	2.60 c	5.67 a	1.67 b	4.00 a	-	7.84 bc
		D25	4.87 a	4.33 c	0.67 e	3.67 ab	-	8.07 bc
		D30	4.70 a	3.67 d	0.67 e	3.00 b	-	10.62 a
	PS-32	D15	1.97 e	3.67 d	2.00 b	1.67 e	-	4.91 d
		D20	2.23 d	4.33 c	1.33 c	2.33 c	0.67a	7.13 c
		D25	3.15 bc	4.00 bc	2.00 b	2.00 d	-	8.71 b
		D30	3.47 b	3.33 e	1.00 d	2.33 c	-	8.35 bc
ANOVA								
C			3.30 ^ns^	8.33 **	1.80 ^ns^	39.20 ***	4.00 ^ns^	0.79 ^ns^
T			10.38 ***	4.56 **	8.73 **	6.93 **	4.00 *	7.02 **
C × T			1.88 ^ns^	8.00 ^ns^	4.47 *	4.00 *	4.00 *	0.73 ^ns^
2022 (Sanya)	YS-25	D15	14.27 e	5.00 c	0.33 c	3.67 c	1.00 f	13.00 e
		D20	16.98 d	7.00 a	0.67 b	4.33 b	2.00 c	15.10 de
		D25	24.60 b	5.00 cd	-	2.67 e	2.33b	23.47 b
		D30	34.08 a	4.00 e	-	1.00 f	3.33 a	27.54 a
	PS-32	D15	7.40 f	5.67 b	2.00 a	2.67 e	1.00 f	13.38 e
		D20	13.35 e	6.67a	-	5.33 a	1.33 d	16.45 d
		D25	19.34 c	4.67 d	-	3.00 d	1.67 cd	16.65 d
		D30	20.53 c	3.67 f	-	1.33 f	2.33 b	19.28 c
ANOVA								
C			4.56 **	10.36 *	1.33 ***	1.50 ^ns^	12.80 **	1.23 ^ns^
T			3.33 *	8.33 **	1.20 ***	49.50 ***	17.33 ***	2.05 **
C × T			2.69 ^ns^	6.23 ^ns^	1.33 ***	4.61 *	1.60 ^ns^	2.45 ^ns^

SR, storage root; Y, year; C, cultivar; T, treatment; YS-25, Yanshu25; PS-32, Pushu32. Two-way ANOVA, LSD was used. Values followed by different letters present significant differences among planting density treatments (*p* < 0.05). * *p* < 0.05; ** *p* < 0.01; *** *p* < 0.001; ^ns^, no significance. ^†^ D15, 15 cm plant distance and 83,280 plants ha^−1^; D20, 20 cm plant distance and 62,520 plants ha^−1^; D25, 25 cm plant distance and 50,025 plants ha^−1^; D30, 30 cm plant distance and 41,640 plants ha^−1^. The row distance was set at 80 cm for all.

**Table 4:** Effect of planting density on root traits during storage root formation (2022 Sanya).

DAP(d)	Cultivar	Treatment ^†^	Adventitious Root Number	Total Root Fresh Weight (g)	Potential SR Diameter (mm)	Potential SR Weight (g)	Potential SR Weight Ratio (%)
15 d	YS-25	D15	13.60 b	2.84 e	1.03 c	1.75 b	61.62 a
		D20	15.20 b	3.44 c	1.16 b	1.96 ab	56.97 ab
		D25	15.20 b	3.69 b	1.12 b	1.89 ab	51.22 b
		D30	13.80 b	4.29 a	1.05 c	2.07 a	48.25 b
	PS-32	D15	17.60 a	2.95 de	1.29 ab	1.64 c	55.59 a
		D20	19.00 a	3.10 d	1.41 a	1.84 ab	59.35 a
		D25	18.20 a	3.64 bc	1.25 ab	1.75 b	48.08 b
		D30	18.00 a	3.80 b	1.26 ab	1.76 b	46.31 b
ANOVA							
C			66.57 ***	12.59 ***	35.50 ***	20.55 ***	6.30 *
T			2.28 ^ns^	81.94 ***	2.86 *	7.38 **	14.15 ***
C × T			0.33 ^ns^	6.21 **	0.67 ^ns^	1.62 ^ns^	1.13 ^ns^
25 d	YS-25	D15	14.00 bc	6.11 b	1.64 c	3.96 a	64.32 a
		D20	15.00 b	6.59 a	2.39 a	3.84 ab	58.27 b
		D25	13.20 c	6.74 a	2.00 b	3.21 c	47.62 c
		D30	14.00 bc	6.94 a	1.96 b	2.75 d	39.62 d
	PS-32	D15	16.60 ab	5.19 c	1.59 c	3.40 b	65.51 a
		D20	17.40 a	5.37 c	2.20 a	3.47 b	64.61 a
		D25	15.40 b	5.41c	2.19 a	3.44 b	63.58 a
		D30	15.20 b	6.18b	2.19 a	3.76 ab	60.84 a
ANOVA							
C			55.98 ***	112.96 ***	1.86 ^ns^	0.61 ^ns^	0.92 ^ns^
T			5.14 **	14.29 ***	16.12 ***	4.61 **	8.51 ***
C × T			0.38 ^ns^	12.04 ***	3.94 *	1.81 ^ns^	5.60 ***
		D15	-	28.90 cd	8.77 c	4.32 c	14.94 c
		D20	-	36.94 b	8.81 c	8.21 a	22.22 a
	YS-25	D25	-	39.83 ab	10.75 b	8.69 a	21.81 a
		D30	-	39.79 ab	9.66 b	7.38 ab	18.54 b
35 d		D15	-	21.19 d	9.27 bc	4.39 c	20.72 a
		D20	-	31.53 c	10.67 b	6.77 b	21.47 a
	PS-32	D25	-	35.80 b	11.13 ab	7.83 ab	21.87 a
		D30	-	41.26 a	13.43 a	7.70 ab	18.66 b
ANOVA							
C			-	2.12 ^ns^	14.91 ***	2.25 ^ns^	0.64 ^ns^
T			-	12.43 ***	6.92 ***	49.01 ***	20.86 ***
C × T			-	2.96 **	2.67 ^ns^	1.64 ^ns^	6.21 **

DAP, days after planting; SR, storage root; Y, year; C, cultivar; T, treatment; YS-25, Yanshu25; PS-32, Pushu32. Two-way ANOVA, LSD were used. Values followed by different letters present significant differences among planting density treatments (*p <* 0.05). * *p <* 0.05; ** *p <* 0.01; *** *p <* 0.001; ^ns^, no significance. ^†^ D15, 15 cm plant distance and 83,280 plants ha^−1^; D20, 20 cm plant distance and 62,520 plants ha^−1^; D25, 25 cm plant distance and 50,025 plants ha^−1^; D30, 30 cm plant distance and 41,640 plants ha^−1^. The row distance was set at 80 cm for all.

**Table 5:** Effect of planting density on dry matter accumulation and allocation during storage root formation (2022 Sanya).

DAP (d)	Cultivar	Treatment ^†^	Total Plant Dry Weight (g)	Root Dry Weight (g)	Shoot Dry Weight (g)	Root Dry Weight Allocation (%)	Shoot Dry Weight Allocation (%)	Root/Shoot Ratio
15 d	YS-25	D15	1.98 c	0.21 d	1.77 d	10.61bc	89.39 a	0.12 b
		D20	2.40 b	0.32 a	2.08 bc	13.33 a	86.67 a	0.15 a
		D25	2.55 a	0.24 c	2.31 a	9.41 cd	90.59 a	0.10 c
		D30	2.56 a	0.28 b	2.28 a	9.94 c	89.06 a	0.11 c
	PS-32	D15	2.04 c	0.23 c	1.86 cd	8.83 d	91.17 a	0.10 c
		D20	2.29 bc	0.31 a	1.98 c	13.54 a	86.46 a	0.16 a
		D25	2.42 b	0.27 b	2.15 b	11.16 b	88.84 a	0.13 b
		D30	2.25 bc	0.28 b	2.05 bc	8.89 d	91.11 a	0.10 c
ANOVA								
C			4.87 *	2.15 ^ns^	6.59 *	10.39 **	8.75 **	11.37 **
T			3.43 *	27.47 ***	2.02 ^ns^	21.20 ***	22.02 ***	19.72 ***
C × T			19.17 ***	1.73 ^ns^	20.93 **	10.25 ***	10.49 ***	8.52 ***
25 d	YS-25	D15	4.23 c	0.62 b	3.61 c	14.80 ab	85.20 a	0.17 b
		D20	4.49 c	0.71 a	3.78 c	15.82 a	84.12 a	0.19 a
		D25	5.06 b	0.67 a	4.39 b	13.00 c	87.00 a	0.15 c
		D30	5.67 b	0.63 b	5.04 a	11.20 d	88.20 a	0.12 d
	PS-32	D15	4.14 c	0.58 c	3.56 c	14.00 b	86.00 a	0.16 bc
		D20	4.27 c	0.61 b	3.66 c	14.20 b	85.80 a	0.16 bc
		D25	4.51 c	0.62 b	3.89 c	11.00 d	89.00 a	0.16 bc
		D30	6.20 a	0.69 a	5.51 a	11.20 d	88.80 a	0.12 d
ANOVA								
C			0.52 ^ns^	5.95 *	0.20 ^ns^	0.64 ^ns^	0.64 ^ns^	1.29 ^ns^
T			47.21 **	4.64 **	46.85 ***	20.81 ***	20.81 ***	21.56 ***
C × T			3.96 *	6.61 ***	3.17 *	2.29 ^ns^	2.29 ^ns^	1.56 ^ns^
		D15	13.44 e	3.58 d	9.88 d	26.60 c	73.40 b	0.36 c
		D20	18.23 c	4.91 c	13.32 c	27.00 c	73.00 b	0.37 c
	YS-25	D25	23.01 b	5.04 c	17.96 b	22.00 d	78.00 a	0.28 d
		D30	25.30a	5.34 bc	19.95 a	21.20 d	78.80 a	0.27 d
35 d		D15	15.34 d	4.76 c	10.08 d	31.00 b	69.00 c	0.47 b
		D20	15.69 d	5.95 b	9.74 d	37.60 a	62.40 d	0.61 a
	PS-32	D25	23.62 b	7.08 a	16.53 b	30.00 b	70.00 bc	0.43 bc
		D30	24.34 ab	6.84 a	17.49 b	28.20 c	71.80 bc	0.39 c
ANOVA								
C			0.51 ^ns^	72.60 ***	24.68 ***	83.33 ***	83.33 ***	28.50 ***
T			208.66 ***	28.38 ***	150.35 ***	16.80 ***	16.80 ***	5.09 **
C × T			7.75 **	1.69 ^ns^	7.11 ***	2.44 ^ns^	2.44 ^ns^	1.67 ^ns^

DAP, days after planting; SR, storage root; Y, year; C, cultivar; T, treatment; YS-25, Yanshu25; PS-32, Pushu32. Two-way ANOVA, LSD was used. Values followed by different letters present significant differences among planting density treatments (*p* < 0.05). * *p* < 0.05; ** *p* < 0.01; *** *p* < 0.001; ^ns^, no significance. ^†^ D15, 15 cm plant distance and 83,280 plants ha^−1^; D20, 20 cm plant distance and 62,520 plants ha^−1^; D25, 25 cm plant distance and 50,025 plants ha^−1^; D30, 30 cm plant distance and 41,640 plants ha^−1^. The row distance was set at 80 cm for all.

**Table 6:** Correlation analysis of storage root number, weight, and yield with gene expression.

	*SuSy*	*SPS*	*SSS*	*AGPase*	*GBSS*	*SBE*	*PAL*	*4CL*
SRN ^†^	0.44	0.61 *^‡^	0.165	0.31	0.72 **	0.30	0.27	0.33
SRFW	−0.39	−0.39	−0.32	−0.23	−0.09	−0.38	−0.26	−0.05
Yield	0.44	0.62 *	0.43	0.29	0.33	0.47	−0.11	0.00
	*C4H*	*CCoAOMT*	*CAD*	*HCT*	*Ibkn1*	*Ibkn2*	*Ibkn3*	-
SRN	−0.20	0.62	0.17	−0.14	0.41	0.39	0.25	-
SRFW	−0.21	−0.41	−0.04	−0.03	−0.35	−0.10	−0.27	-
Yield	−0.24	0.16	−0.43	−0.25	0.51	0.08	0.32	-

^†^ SRN, storage root number per plant; SRFW, storage root fresh weight. ^‡^, * and ** indicate significant differences at *p* < 0.05 and *p* < 0.01, respectively.

## Data Availability

All data are included in the present study.

## Supplementary Materials

The following supporting information can be downloaded at: https://www.mdpi.com/article/10.3390/plants12102039/s1, Table S1: Climatic conditions; Table S2: Experimental soil physical and chemical properties; Table S3: Gene primer sequences used in qRT-PCR.
