# Supplementary material for: Influence of Planting Density on Sweet Potato Storage Root Formation by Regulating Carbohydrate and Lignin Metabolism
Source: Plants (Basel). 2023 May 19;12(10):2039. doi: 10.3390/plants12102039 (PMC10221243; doi:10.3390/plants12102039)
Supplement: Supplementary file 1 [file plants-12-02039-s001.zip › plants-2268713-supplementary.pdf]

plants

Table S1. Climatic growth condition for sweet potato.

Year	Month	Mean temperature (°C)	Precipitation (mm)	Field irrigation (mm)
2020	Nov.(Haikou)	23.40	14.80	100
	Dem.	19.07	6.20	
	Jun.	16.35	19.10	
	Few.	19.81	26.20	
2021	Mar.	24.20	2.00	
	Nov. (Sanya)	26.65	10.20	100
	Dem.	24.84	8.30	
	Jun.	22.56	15.60	
2022	Few.	24.36	14.30	
	Mar.	25.89	6.40	

Table S2. Experimental soil physical and chemical properties.

Experimental sites	Sand content (%)	Soil density (g·cm$^{-2)}$	pH	Organic matter content (%)	Alkalihydrolysable nitrogen (mg·kg^{-1})	Available phosphorus (mg·kg^{-1})	Available potassium (mg·kg^{-1})
Haikou	50.4	1.41	6.84	1.21	52.56	9.01	80.63
Sanya	48.3	1.40	6.77	1.47	74.09	14.36	69.82

Table S3. Genes primer sequences used in qRT-PCR.

Gene	*Forward(5′-3′)*	*Reverse(3′-5′)*
PAL	GGATCCAAGAGTGCAGGTCC	CCTTGTCACATTCCTCCCCG
C4H	GCGGCAAGAAGTACAAGCTC	CTTGGCGTAATCGGTGAGAT
4CL	CTGAGGATGAAGTTAAAGAGTTTGTG	GCCTGAGGGAGACTTTGGA
HCT	CCGTCGCTTACAGCTCCTAC	CGGTGGCTATGTACAGCTTG
CCoAOMT	GAGGCACCCACAAGACTACG	TGGTTGTCTGATTCTCCGCC
CAD	GTCTTGGCGCAGACTCTTTC	TAATGGCACAACAGCGTGAT
Ibkn1	AAAACTCGGGAGATCACTGC	GCAAGCCTCCTCCAATCTC
Ibkn2	GCCAGGCAGAAGTTGCTTAG	CAGTGCCGTTTTCTTTGGTT
Ibkn3	CGCCTAGGTCCATAATCC	TATTTCAAGGCGGTCTCA
GBSS	GGACGTGCTGAAGGTGATAA	CGAGAGCTCTTGTGACATGC
Sps	TCTGAAGATTTCTCGGATGA	AGTAAGGAGCATAGGCACA
Susy	CTTGAGATTCGTCGCTACCTT	CTGAACCCTCCCTTCATCTTAC
AGPase	GCAGACTTGTCTAGATCCTGATG	CCGCTTCTTTGTGAGAGGATAG
SSS	GACTGTGGGATCTACTGAAAGG	TTGCTGGCTCCTGAGAATTTA
SBEI	CCTTCTCGTGGGTCTTTCATAC	ACCAGTAGTGCATGGTGAAG
β-actin	AGCAGCATGAAGATTAAGGTTGTAGCAC	TGGAAAATTAGAAGCACTTCCTGTGAAC
